# Exploratory study to evaluate two clinical methods for assessing moisturizing effect on skin barrier repair

**DOI:** 10.1111/srt.12632

**Published:** 2019-04-02

**Authors:** Jane Snatchfold, Darren Targett

**Affiliations:** ^1^ CK Clinical Chesterfield UK; ^2^ Primoris Contract Solutions Ltd Ascot UK

**Keywords:** barrier repair, dry skin, exploratory method, moisturizer, skin barrier

## Abstract

**Background:**

Two clinical methods of assessing a moisturizer's effect on stratum corneum (SC) barrier repair were evaluated in female subjects with dry skin, to identify an assessment method for future studies.

**Methods:**

In this single‐centre, split‐body study, women with dry skin applied moisturizer before (method A) or after (method B) SC barrier perturbation using D‐Squame® stripping discs. Transepidermal water loss (TEWL) and residual protein on D‐Squame discs were assessed over 14 days.

**Results:**

Twenty‐four subjects were included. For method A, the mean slope values of plots of 1/TEWL vs cumulative protein removed decreased over time for both treated and untreated areas, indicating improved SC barrier quality. There were no significant differences between treated and untreated areas, although a trend to a more negative slope was observed by Day 14 in the treated areas (*P *=* *0.082), suggesting treatment improved barrier quality. For method B, using pre‐ and post‐stripping as covariates, no statistical differences/trends were observed between treated and untreated areas for change in TEWL from post‐stripping to any evaluation from Days 3‐14. TEWL values returned towards pre‐stripping values for treated and untreated areas by the initial Day 3 evaluation.

**Conclusion:**

For method A, there were trends suggesting the moisturizing treatment improved SC barrier quality. For method B, there were no significant differences/trends between treated and untreated areas. Further assessment with different methodologies is warranted to design appropriate clinical protocols for evaluating accelerated skin barrier repair. These data are insufficient to conclude whether the product or methodology was responsible for the results.

## 1 INTRODUCTION

The stratum corneum (SC), the outermost layer of the skin, is the main permeability barrier of the skin.^1^ It protects the underlying tissue from water loss, chemicals, infection and mechanical stress.^1^, ^2^ Transepidermal water loss (TEWL) is commonly used to measure SC barrier function in the clinical setting^1^; however, TEWL measurements alone provide only an apparent measure of barrier quality (ie, the skin's overall resistance to passive water diffusion through the skin).^3^

Recently, Lu and colleagues developed a novel clinical and data analysis procedure based on sequential tape stripping with TEWL measurement and SC protein analysis, in the course of a study investigating the SC barrier and the hygroscopic properties of normal and cosmetic dry skin. This procedure was thought to be a more robust method than those previously used for SC barrier analysis.^3^

Building on the findings of Lu et al,^3^ the present study explored two different clinical methods of assessing the effect of a moisturizing product on SC barrier repair in female subjects with dry skin, with the objective of identifying an assessment method for accelerated barrier repair for use in future studies.

## 2 MATERIALS AND METHODS

### 2.1 Study design

This was a single‐centre, non‐randomized, split‐body study to explore two clinical methods of assessing SC barrier repair, using a marketed cosmetic moisturizer (Curel, a registered trademark of Kao Corporation, Tokyo, Japan) containing glycerine, isopropyl palmitate, petrolatum, and *Butyrospermum parkii* (shea) butter in female subjects with dry skin. The primary objective was to identify an accelerated method of assessing SC barrier repair. The study was conducted between 8 April and 22 May 2015 at a single centre in Irving, TX, USA, in accordance with applicable local ethical and regulatory requirements. All subjects provided written informed consent.

### 2.2 Subjects

Eligible subjects were healthy female Caucasians, aged ≥18 years, with a minimum dryness grading of 2 (moderate flaking/scaling; 0‐4 grading scale) at baseline (Day 0) on both lower legs and ≤0.5‐point difference between the right and left leg and the upper and lower areas on each outer leg, as determined by the visual grading of a trained examiner.

### 2.3 Methods and assessments

Eligible subjects underwent a 7‐day washout period, using only the provided soap to cleanse the lower legs (Ivory® Original; Procter & Gamble, Cincinnati, OH, USA). This soap was used from screening until study completion.

The intervention included two methods that were defined as method A and method B. A subject's right lower leg was used for method A, and a subject's left lower leg was used for method B. For both methods, subject visits/assessments took place at baseline and on Days 3, 5, 7, 10, 12 and 14.

At the baseline visit (Day 0), the outer aspects of a subject's lower legs were each marked into an upper test area to be treated with moisturizer and a lower test area to be left untreated.

For method A, seven small areas were marked on the upper area (#1‐7) and seven on the lower area (#8‐14) where stripping (using D‐Squame® stripping discs; CuDerm Corporation, Dallas, TX, USA) and TEWL measurement were taken. At baseline and at each subsequent visit, TEWL was measured at designated times (after 5, 10, 15 and 20 tape strips) on two of the smaller marked skin areas (one treated, the other untreated; all different at each visit). Residual proteins on the D‐Squame discs were also measured.

For method B, two small areas were marked, one on the treated upper area (#15) and one on the untreated lower area (#16) where the stripping and TEWL measurements were taken. At baseline visit, TEWL measurements were taken at the designated skin areas before and after skin barrier perturbation, with D‐Squame® stripping discs applied and removed 20 times. No skin stripping was performed on the left leg during subsequent visits, only TEWL was measured.

Following baseline assessments, subjects were instructed to apply 240 mg moisturizer (approximately 2 mg/cm^2^ equivalent) to the marked upper area of both legs twice daily (morning and evening) for 14 days.

Subjects were acclimatized for 30 minutes before trained examiner visual grading of dryness (baseline visit) and TEWL measurements were performed. There was no moisturizer application prior to the morning visits (on Days 3, 5, 7, 10, 12 and 14).

For both methods, moisturizer was applied 30 minutes after the last TEWL measurement on the upper test area only. The last moisturizer application was on the evening before the Day 14 visit.

Transepidermal water loss assessments for this study were performed using the Tewameter® TM 300 (Courage + Khazaka electronic GmbH, Cologne, Germany) device. D‐Squame disc protein mass was measured using the SquameScan® 850 (Heiland electronic GmbH, Wetzlar, Germany). Safety was assessed throughout the study by monitoring of adverse events (AEs).

### 2.4 Statistical analyses

As described in the method of Lu et al,^3^ the relative barrier quality among the subjects (or subject groups) can be obtained by comparing the slopes of the regression of the 1/TEWL vs cumulative protein (C*p*) removed for each subject (or subject group). The relative SC thickness can be determined from the values of C*p* removal that can be quantified according to where the regression lines intercept the *x*‐axis. It was planned to recruit 25 subjects so that at least 20 evaluable subjects would complete the study. With 20 subjects, the study had 90% power to show a difference between treated and untreated groups of 32% in SC barrier quality and 22% in SC thickness. This assumed a standard deviation of the paired differences (treated minus untreated) of 1.4 × 10^−5^ for slope of 1/TEWL vs C*p* removed and 1150 μg for total protein removed.

Due to the exploratory nature of the study, a number of different parameters were evaluated in various ways to try to ascertain the most appropriate measure(s) of barrier repair for use in future studies.

For method A, a random coefficients model was fitted with 1/TEWL as the response variable and fixed‐effect model terms for C*p*, treatment (treated or untreated), and the protein*treatment interaction and random coefficients for subject*treatment and the subject*treatment*protein interaction. At each visit, values for C*p* were obtained before tape stripping (C*p *= 0), and after 5, 10, 15 and 20 strips were removed. For each individual subject, the slope of the 1/TEWL vs C*p* was obtained to demonstrate the point at which the regression line intercepts the C*p* axis, to be able to determine SC quality and thickness. For each post‐baseline visit, the change in TEWL value from pre‐ to post‐stripping (after removal of 20 strips) was calculated and compared between treated and untreated areas. A repeated measures model was fitted with factors for treatment, day and treatment*day (fixed effects), and subject (random effect), and a covariate for change from pre‐ to post‐stripped TEWL assessed at baseline.

For method B, the change in TEWL value from post‐stripping at baseline to each subsequent visit was calculated and compared between treated and untreated areas. A repeated measures model (analysis model 1) was fitted with factors for treatment, day and treatment*day (fixed effects), and subject (random effect), and covariates for pre‐ and post‐stripped TEWL (both measured at baseline). An alternative exploratory model (analysis model 2) was fitted using only a single covariate of change from pre‐ to post‐stripped TEWL assessed at baseline.

## 3 RESULTS AND DISCUSSION

### 3.1 Subjects

Of 28 subjects screened, 24 participated in the study and were included in the intent‐to‐treat and safety populations. All were female, Caucasian, and had a leg dryness grading at baseline of ≥2. The mean (standard deviation) age was 39.6 (9.87) years and the age range was 20‐55 years.

### 3.2 Method A

**Figure 1:**
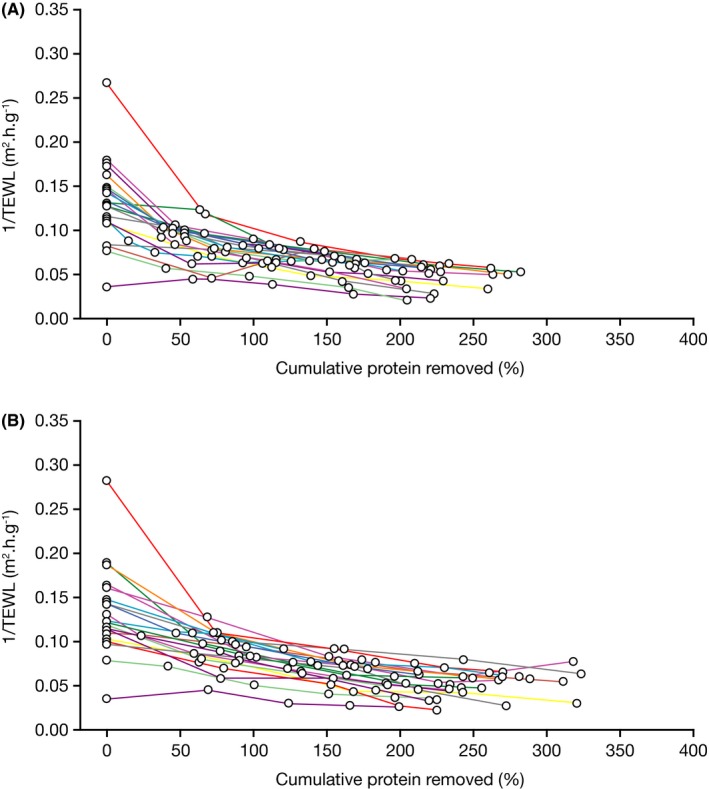
Individual 1/TEWL vs cumulative protein removal on Day 14 for (A) treated and (B) untreated subjects. TEWL, transepidermal water loss

**Figure 2:**
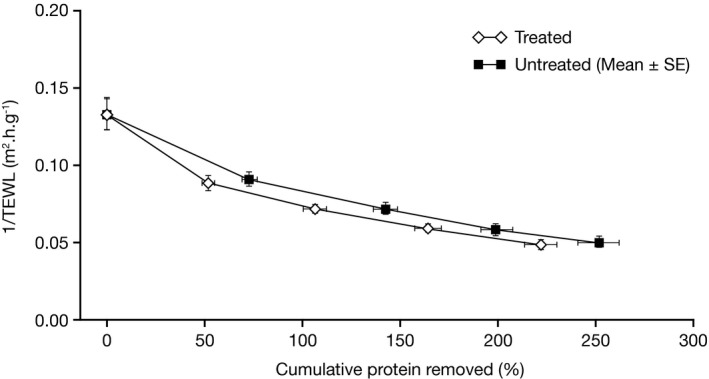
Mean 1/TEWL vs cumulative protein removal on Day 14. Values represent mean (±SE). SE, standard error; TEWL, transepidermal water loss

**Figure 3:**
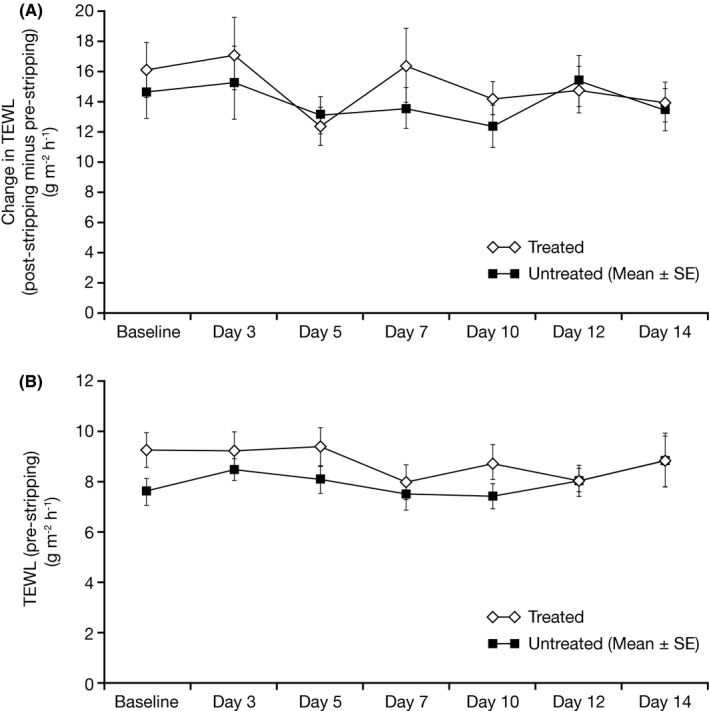
Method A‐(A) Mean (±SE) change in TEWL values from pre‐ to post‐stripping by visit day, and (B) Mean (±SE) pre‐stripping TEWL values by visit day, across the 14‐day study period. SE, standard error; TEWL, transepidermal water loss

The mean slope values of plots of 1/TEWL vs C*p* decreased over time for both treated and untreated areas, indicating an improvement in SC barrier quality (Figure 1 and Figure 2). Slope values were not significantly different between treated and untreated areas at any time; however, a trend was observed for values of treated areas to be more negative than untreated areas by Day 14 (*P *=* *0.082) (Table 1a). The total amount of protein removed by tape stripping on untreated areas was higher than for the treated areas (with statistically significant differences seen at Days 7, 12 and 14; Table 1b). This could possibly be attributable to the fact that moisturization improves the skin dryness, which could therefore reduce the amount of cells (proteins) taken off during stripping. Values for change in TEWL from pre‐ to post‐stripping were not significantly different between treated and untreated areas at any visit, and no trend was observed (Table 1c and Figure 3A). Values for change in TEWL from pre‐stripping at baseline to pre‐stripping at each visit were generally not significantly different over the course of the study for both treated and untreated areas (Table 1d and Figure 3B). These findings suggest that the moisturizer treatment did not produce physiological improvement in the SC barrier under the conditions of this study.

**Table 1:** Method A: Comparison between treated and untreated sites by visit day over the 14‐day study period

Visit	Treated (N = 24) LS mean	Untreated (N = 24) LS mean	% difference	Difference LS mean	95% CI	*P*‐value
(a) Slope (relative barrier quality) (m^2^/h/g/%^3^)						
Day 3	−0.280	−0.279	−0.4	−0.001	−0.047 to 0.045	0.956
Day 5	−0.285	−0.309	7.5	0.023	−0.038 to 0.084	0.445
Day 7	−0.368	−0.353	−4.2	−0.015	−0.088 to 0.058	0.676
Day 10	−0.321	−0.324	0.8	0.002	−0.055 to 0.059	0.930
Day 12	−0.364	−0.324	−12.1	−0.039	−0.093 to 0.014	0.142
Day 14	−0.346	−0.302	−14.4	−0.043	−0.093 to 0.006	0.082
(b) Total SC protein (relative SC thickness) (%)						
Day 3	430	421	2.1	9	−48 to 66	0.755
Day 5	419	426	−1.7	−7	−48 to 33	0.711
Day 7	363	416	−12.8	−53	−87 to −19	**0.003**
Day 10	390	419	−6.9	−29	−60 to 2	0.068
Day 12	347	381	−8.8	−34	−56 to −11	**0.005**
Day 14	350	418	−16.2	−68	−95 to −41	**< 0.001**
(c) Change in TEWL (pre‐ to post‐stripping) (g/m^2^/h)						
Day 3	16.74	15.25	9.7	1.49	−4.05 to 7.02	0.587
Day 5	12.39	13.82	−10.3	−1.43	−3.89 to 1.03	0.239
Day 7	16.54	14.55	13.7	1.99	−3.19 to 7.17	0.442
Day 10	14.26	13.18	8.2	1.08	−1.60 to 3.77	0.414
Day 12	14.88	16.35	−8.9	−1.46	−5.44 to 2.51	0.459
Day 14	13.85	13.90	−0.4	−0.05	−3.92 to 3.82	0.978
(d) Change in TEWL (baseline to pre‐stripping) (g/m^2^/h)						
Day 3	0.846	0.194	336.2	0.652	−1.090 to 2.393	0.450
Day 5	0.797	−0.361	320.8	1.158	−0.806 to 3.122	0.237
Day 7	−0.580	−0.946	38.7	0.366	−0.793 to 1.525	0.520
Day 10	0.235	−1.014	123.2	1.250	0.202 to 2.297	**0.021**
Day 12	−0.499	−0.416	−19.8	−0.083	−1.133 to 0.968	0.870
Day 14	0.351	0.325	7.8	0.025	−2.040 to 2.091	0.980

(a) Slope values of plots of 1/TEWL vs *Cp* at each visit day; (b) Total amount of SC protein removed by tape stripping at each visit day; (c) Change in TEWL values from pre‐ to post‐stripping by visit day; and (d) Change in TEWL values from pre‐stripping at baseline to pre‐stripping at each visit day.

Significant *P*‐values are shown in bold.

CI, confidence interval; LS, least squares; SC, stratum corneum; TEWL, transepidermal water loss.

### 3.3 Method B

**Figure 4:**
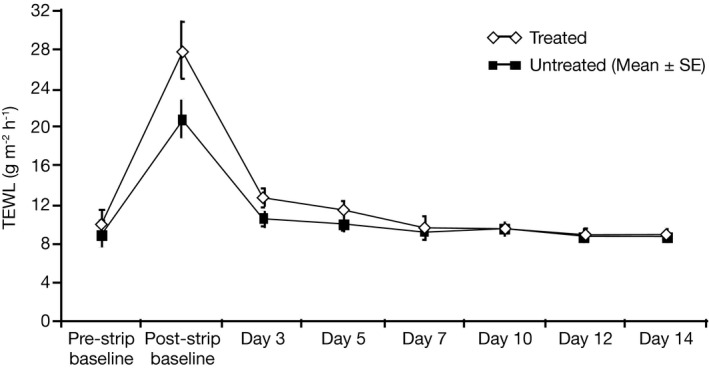
Method B‐Change in mean (±SE) TEWL values across the 14‐day study period. SE, standard error, TEWL, transepidermal water loss

Transepidermal water loss values for both treated and untreated areas decreased over time from the post‐stripping value at baseline, indicating improved SC barrier repair. Analysis model 1, using both pre‐ and post‐stripping TEWL baseline values as covariates, generally showed no significant difference between treated and untreated areas. The TEWL values for treated and untreated areas decreased to a similar extent over the study period, demonstrating that both areas eventually return to values measured at baseline (Table 2a and Figure 4). Exploratory analysis model 2, using a single covariate of change in TEWL from pre‐ to post‐stripping at baseline, showed a decrease in TEWL over the study period for both treated and untreated areas, with the magnitude of the decrease being greater for the treated sites at Days 3‐14 and statistically significant differences seen at Days 10 and 14 (Table 2b and Figure 4). This suggests a greater degree of SC barrier repair for treated areas compared with untreated areas.

**Table 2:** Method B‐Change in TEWL values from post‐stripping at baseline over the 14‐day study period using (a) Analysis model 1, or (b) Exploratory analysis model 2

Visit	Treated (N = 24) LS mean	Untreated (N = 24) LS mean	% difference	Difference LS mean	95% CI	*P*‐value
(a) Change in TEWL from post‐stripping at baseline (g/m^2^/h)‐analysis model 1						
Day 3	−10.76	−12.50	13.9	1.74	0.10 to 3.37	**0.038**
Day 5	−11.96	−13.13	8.9	1.17	−0.41 to 2.74	0.142
Day 7	−13.73	−13.98	1.8	0.25	−1.87 to 2.37	0.815
Day 10	−13.77	−13.72	−0.4	−0.05	−1.15 to 1.05	0.925
Day 12	−14.46	−14.48	0.1	0.02	−1.25 to 1.29	0.977
Day 14	−14.39	−14.45	0.4	0.06	−1.53 to 1.65	0.941
(b) Change in TEWL from post‐stripping at baseline (g/m^2^/h)‐exploratory analysis model 2						
Day 3	−11.93	−11.41	−4.5	−0.51	−2.51 to 1.48	0.596
Day 5	−13.15	−11.88	−10.6	−1.26	−4.05 to 1.52	0.355
Day 7	−14.87	−12.80	−16.1	−2.06	−4.77 to 0.65	0.130
Day 10	−14.92	−12.50	−19.3	−2.42	−4.77 to −0.06	**0.045**
Day 12	−15.63	−13.26	−17.9	−2.37	−4.82 to 0.09	0.058
Day 14	−15.48	−13.32	−16.2	−2.16	−4.19 to −0.13	**0.039**

Significant *P*‐values are shown in bold.

CI, confidence interval; LS, least squares; TEWL, transepidermal water loss.

### 3.4 Safety

One AE of dermatitis following contact with poison ivy was reported; this was mild and not considered to be related to the study product. No serious AEs were reported.

## 4 CONCLUSIONS

Although the study by Lu and colleagues demonstrated a method that was thought to be more robust than those previously used for SC barrier analysis,^3^ the present investigation did not manage to secure a significant exploratory method for the purposes of accelerated repair.

Findings from method A suggest that the moisturizing treatment, while appearing to improve apparent SC barrier quality and SC thickness, did not produce accelerated physiological improvement in the SC barrier under the conditions of this study. Findings from method B, using both analysis methods, demonstrated a decrease in TEWL over time from the post‐stripping value at baseline for both the treated and untreated areas, indicating SC barrier repair over the course of the study.

The results of this study precluded validation of the accelerated barrier repair methodology. In addition, it was not possible to determine whether the product or the methodology was responsible for the results. Further exploratory work would be required to develop and validate a clinical method to assess accelerated skin barrier repair.

## CONFLICT OF INTEREST

Jane Snatchfold provides independent consulting services to GlaxoSmithKline Consumer Healthcare. Darren Targett provided independent consulting services to GlaxoSmithKline Consumer Healthcare at the time the analysis was conducted.
